# Efficacy of chemotherapy for lymph node-positive luminal A subtype breast cancer patients: an updated meta-analysis

**DOI:** 10.1186/s12957-020-02089-y

**Published:** 2020-12-02

**Authors:** Yilun Li, Li Ma

**Affiliations:** Hebei Medical University Fourth Affiliated Hospital and Hebei Provincial Tumor Hospital, Shijiazhuang, China

**Keywords:** Luminal A, Lymph node-positive breast cancer, Prognosis, Meta-analysis

## Abstract

**Objective:**

To assess the association between chemotherapy and prognosis of patients with breast cancer of luminal A subtype and lymph node-positive, luminal A subtype breast cancer

**Methods:**

Articles published between January 1, 2010, and May 1, 2020, were collected from PubMed, Embase, and Web of Science databases. On the basis of a test for heterogeneity, we selected the random effects model or fixed effects model for meta-analysis. Article quality was evaluated by sensitivity analysis, and Begg’s and Egger’s tests were used to measure publication bias.

**Results:**

Six eligible articles were identified. The hazard ratio of overall survival of luminal A breast cancer patients who received both chemotherapy and endocrine therapy was 1.73 (95% CI 1.23, 2.43). The hazard ratio of overall survival for lymph node-positive, luminal A breast cancer patients who received chemotherapy and endocrine therapy was 1.86 and 95% CI 1.26, 2.81. The hazard ratio of relapse-free survival to disease-free survival was 1.30 (95% CI 0.85, 1.77). Tumor size, vascular invasion, and age did not show significant correlations with breast cancer prognosis.

**Conclusion:**

Compared with endocrine therapy alone, the addition of chemotherapy did not improve the prognosis of patients with luminal type A and lymph node positive cancer; instead, side effects of the additional chemotherapy may have negatively affected prognosis. Prospective studies are needed to determine whether the number of positive lymph nodes also correlates with efficacy of chemotherapy of luminal type A breast cancer.

## Introduction

Breast cancer is one of the most common cancers of women. In 2019, breast cancer accounted for 30% of female tumors, ranking first in morbidity; mortality was second for female tumors, accounting for about 26.8% [1].

To facilitate identification and treatment of breast cancer with different characteristics, investigators have classified tumors by subgroup according to the expression patterns of genes. This approach has technical limitations, and high cost prevents its wide use. Instead, clinicians more generally use immunohistochemical markers to classify tumors into subtypes [2]. The molecular subtypes recognized immunologically are the following: luminal A, luminal B, basal-like, and HER2 (human epidermis growth factor receptor-2). Molecular typing is related to the clinicopathological and prognostic characteristics of patients [3].

Luminal type A has better prognosis than other breast cancer subtypes. Oncologists define luminal A as estrogen receptor (ER) > 1%, progesterone receptor (PR) ≥ 20%, breast cancer with negative human epidermal growth factor receptor-2 (HER2), and Ki-67 < 14% of clinical cases [4]. Luminal A has high hormone receptor expression, negative HER2 expression, and a low proliferation rate, properties that contribute to its better prognosis [5].

There are three principal treatments for breast cancer: drug treatment, radiotherapy, and surgery. Drug treatment may also include endocrine therapy, drugs that target the HER2 receptor, and chemotherapy. For luminal type A, endocrine therapy is often more applicable [6]. However, there is still controversy about whether oncologists should include chemotherapy for luminal A, lymph node-positive breast cancer. Because lymph node-positive is a high-risk factor, breast cancer patients with positive luminal type A lymph node can benefit from chemotherapy [7, 8]. Other studies have failed to show benefit from chemotherapy for patients with luminal type A. Even in the case of positive lymph nodes, chemotherapy has not improved prognosis significantly [9, 10]. Therefore, it remains to be determined whether chemotherapy has clinical significance for luminal A type, especially for patients with positive lymph nodes. Coates et al. suggested that luminal A requires chemotherapy only when the number of positive lymph nodes is ≥ 4 [11]. The National Comprehensive Cancer Network guidelines recommend that patients with luminal A breast cancer and patients with positive lymph nodes should receive chemotherapy regardless of the number of nodes [12]. Considering this disparity in recommendations, and to identify factors that may affect prognosis, we have analyzed studies of lymph node-positive luminal A breast cancer patients who received chemotherapy. This analysis will provide ideas for clinical treatment plans and assessment of prognosis.

## Methods

### Search strategy

We searched the PubMed, Embase, and Web of Science databases for research articles published between January 1, 2010, and May 1, 2020. Keywords used in the search were the following: “luminal A”, “breast cancer”, “chemotherapy”, and “lymph node positive”. Eligible research articles must meet the following criteria: (1) research for breast cancer; (2) must include patients with breast type; (3) treatment methods must include chemotherapy and nonchemotherapy; (4) data must include either patient overall survival (OS), relapse-free survival (RFS), or disease-free survival (DFS). The indicator must be either relative risk (RR), odds ratio (OR), or risk ratio (RR) and contain the corresponding 95% confidence interval (CI). OS was defined as the time from the date of diagnosis of breast cancer to the time of death from any cause. The RFS/DFS ratio was defined as the time from the first treatment to progression, recurrence, and death of a patient with breast cancer. For meta-analysis, we used the principles of Preferred Reporting Items for Systematic Reviews and Meta-Analyses (PRISMA).

### Data extraction

The following information was extracted from each article included in the study: first author’s surname, study design, case origin or country in which the study was conducted, follow-up time, number of subjects, type of chemotherapy regimen, assessment outcome indicators and corresponding RR, OR, or HR, and 95% confidence interval. For each study, the treatment group that received only endocrine therapy was set as a reference (nonchemotherapy group). The chemotherapy group referred to the group that received both chemotherapy and endocrine therapy. Chemotherapy was used as the exposure factor. If the articles were grouped according to factors, such as the number of positive lymph nodes, the confidence interval and effect size (relative risk, odds ratio, or hazard ratio) in the groups were also extracted. We stored all extracted information in a well-structured table.

### Statistical analysis

We extracted the RR/HR/OR about the prognostic index of luminal A breast cancer from each study. Risk ratio and hazard ratio were regarded as equivalent measures of risk, and the odds ratio value was regarded as the hazard ratio [13, 14]. The heterogeneity of effects between different studies was assessed by an *I*^2^ statistical test or Q test, in which *I*^2^ < 50 or *P* < 0.1 of Q test indicated significant heterogeneity [15]. We selected the fixed effect model for meta-analysis according to the results of the test for heterogeneity. We also performed a sensitivity analysis by excluding one study at a time to determine whether the results changed significantly. In addition, we conducted a subgroup analysis to verify the influence of various factors on the relationship between chemotherapy and luminal type A breast cancer in the subgroup analysis. Lastly, we assessed publication bias by using Begg’s funnel plot and Egger’s linear regression. All statistical analyses were performed with Stata 14.0 (StataCorp, College Station, TX, USA).

## Results

### Literature search and study characteristics

**Fig. 1:**
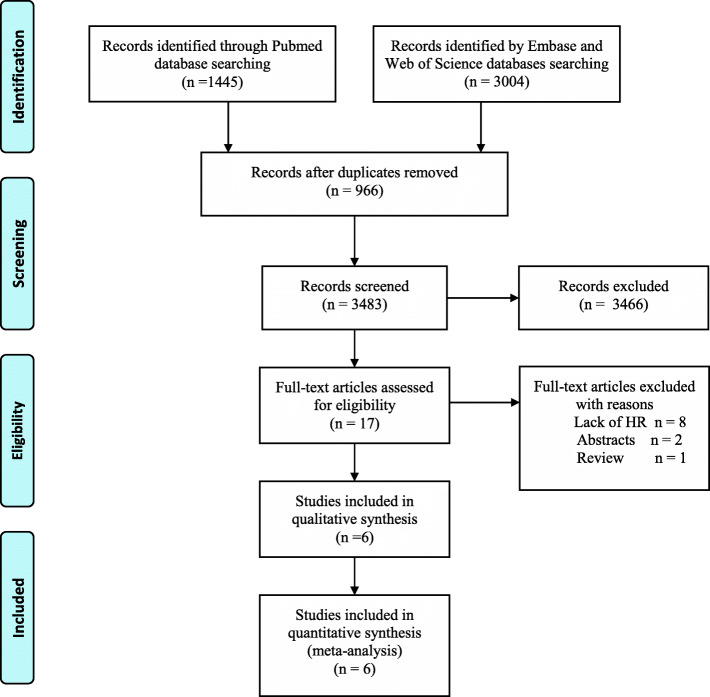
A Preferred Reporting Items for Systematic Reviews and Meta-Analyses (PRISMA) flow chart of the selection procedure of studies to assess the relationship between chemotherapy and prognosis of luminal A breast cancer

We used the keywords “luminal A”, “breast cancer”, “lymph node positive”, and “chemotherapy” to search the PubMed, Embase, and Web of Science databases. From 4449 articles, we removed 966 duplicates, and 3466 articles were excluded after reviewing the title or abstract (Fig. 1). We read the full text of 17 articles and finally included six articles [10, 16–20]. Table 1 shows the details of these publications.

**Table 1:** Characteristics of articles in the meta-analysis on chemotherapy and luminal A breast cancer

Author, year	Country, follow-up (median)	Study design	Sample size	Chemotherapy regimen	Outcome indicators
Uchida et al.2013 [10]	Japan, 3–113 (40) months	Retrospective study	140	FEC × 4/FEC × 4-docetaxel × 4 or paclitaxel × 4	RFS, OS
Kwak et al. 2015 [16]	South Korea, 28–116 (61) months	Retrospective study	879	No detail description	DFS, OS
Han et al. 2015 [17]	China, 60–89 (67) months	Retrospective cohort study	1580	AC × 4/AC × 6	DFS, OS
Nielsen et al. 2016 [18]	UK, 10 years (median)	Clinical trial	1072 (luminal A *n* = 165)	C × 12/CMF × 12	DFS, OS (not provided)
Alramadhan et al. 2016 [19]	South Korea, 51.3 ± 18.9(median)	Retrospective study	406	AC × 4/FAC × 4	DFS, OS (not provided)
Herr et al. 2019 [20]	Multicenter, -	Retrospective multicenter Study	1376	A/AT	RFS, OS

*F* fluorouracil, *E* epirubicin, *C* cyclophosphamide, *A* anthracycline, *M* methotrexate; *T* Taxane, *RFS* relapse-free survival, *OS* overall survival, *DFS* disease-free survival

### Summary hazard ratio for chemotherapy versus nonchemotherapy for luminal A breast cancer

**Fig. 2:**
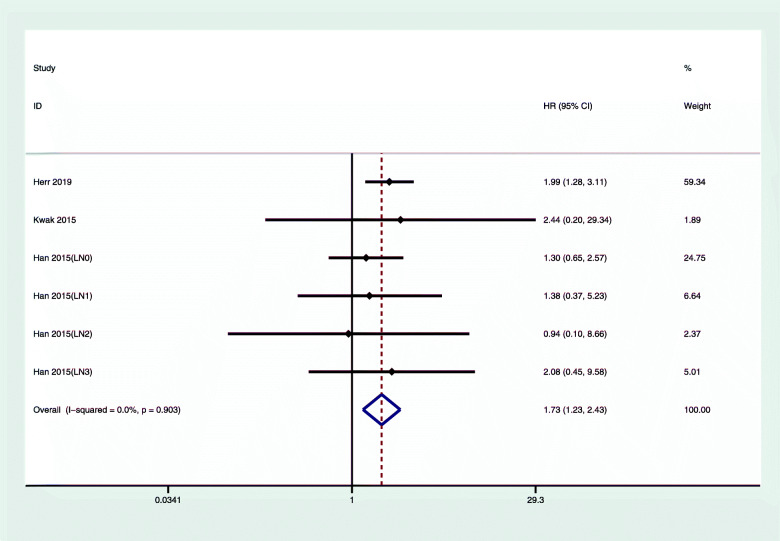
Multivariate-adjusted OS of luminal A breast cancer with chemotherapy versus nonchemotherapy. CI, confidence interval; HR, hazard ratio, OS, overall survival

**Fig. 3:**
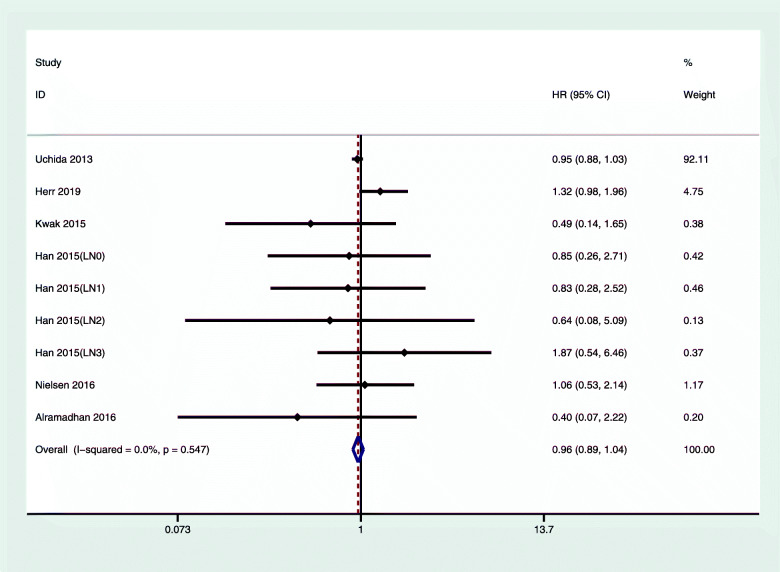
Multivariate-adjusted DFS/RFS of luminal A breast cancer with chemotherapy versus nonchemotherapy. CI, confidence interval; HR, hazard ratio; RFS, relapse-free survival; DFS, disease-free survival

Figure 2 shows the comparison between chemotherapy and nonchemotherapy treatment. Compared with the nonchemotherapy group, the hazard ratio of OS in luminal type A breast cancer for patients who received chemotherapy was 1.73 (95% CI 1.23, 2.43); we did not observe any obvious heterogeneity (*I*^2^ = 0%, *P* = 0.903). Figure 3 shows that the overall DFS/RFS hazard ratio of luminal type A breast cancer in the chemotherapy group and the nonchemotherapy group was 0.86 (95% CI 0.89, 1.04); we did not observe any obvious heterogeneity.

### Summary hazard ratio for chemotherapy versus nonchemotherapy for lymph node-positive luminal A breast cancer

**Fig. 4:**
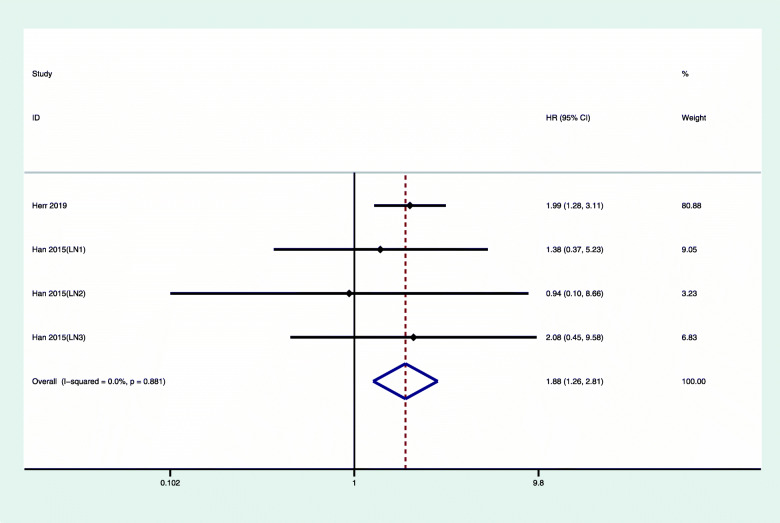
Multivariate-adjusted OS of luminal A, lymph node-positive breast cancer with chemotherapy versus nonchemotherapy. CI, confidence interval; HR, hazard ratio, OS, overall survival

**Fig. 5:**
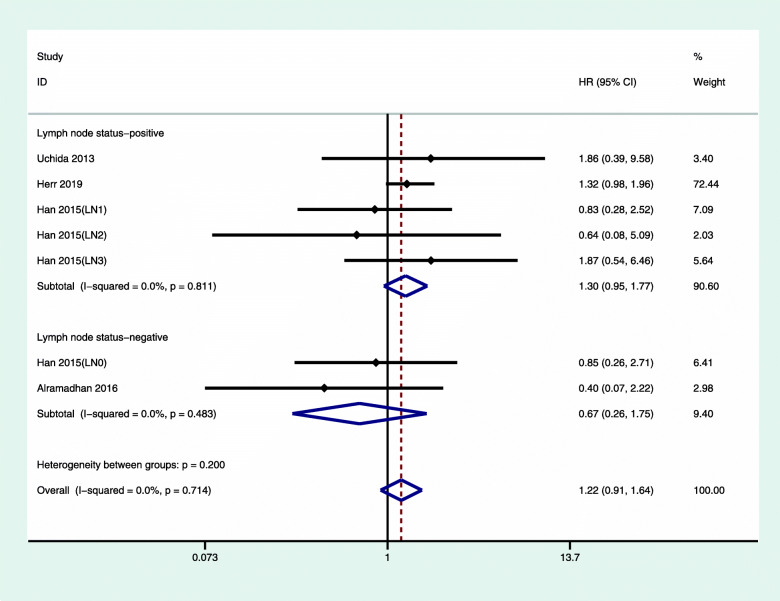
Multivariate-adjusted DFS/RFS of luminal A breast cancer with chemotherapy versus nonchemotherapy, sorted by lymph node status. CI, confidence interval; HR, hazard ratio; RFS, relapse-free survival; DFS, disease-free survival

We also analyzed the relationship between chemotherapy and the prognosis of patients with luminal A type breast cancer with positive lymph nodes (Fig. 4). The overall hazard ratio of OS for lymph node-positive luminal A with chemotherapy was 1.86 (95% CI 1.26, 2.81). We did not observe any obvious heterogeneity (*I*^2^ = 0%, *P* = 0.881). Figure 5 shows that the overall hazard ratio of DFS/RFS for lymph node-positive breast cancer treated with chemotherapy was 1.30 (95% CI 0.85, 1.77). For luminal type A, lymph node-negative breast cancer, the overall hazard ratio of DFS/RFS was 0.67, (95% CI 0.85, 1.77), and there was no obvious heterogeneity.

### Subgroup analysis of other factors

**Fig. 6:**
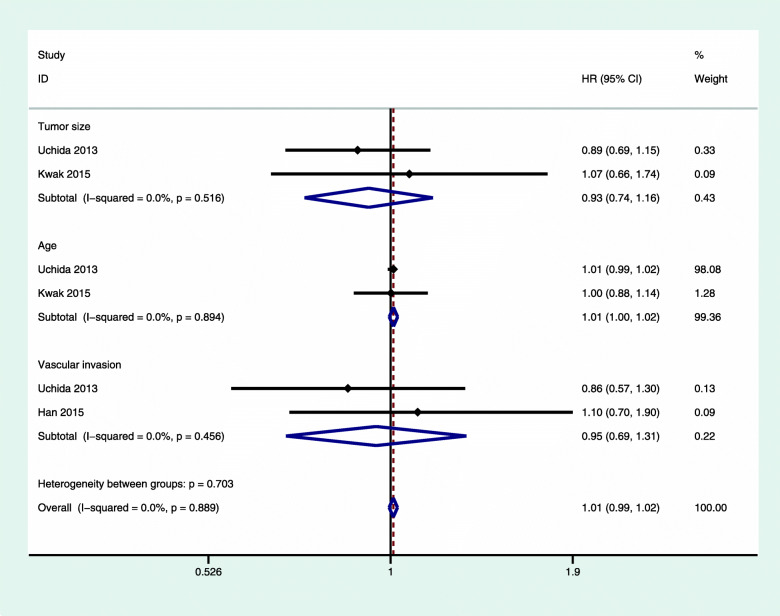
Subgroup analysis of OS of luminal A breast cancer with chemotherapy versus nonchemotherapy. OS, overall survival; CI, confidence interval; HR, hazard ratio

**Fig. 7:**
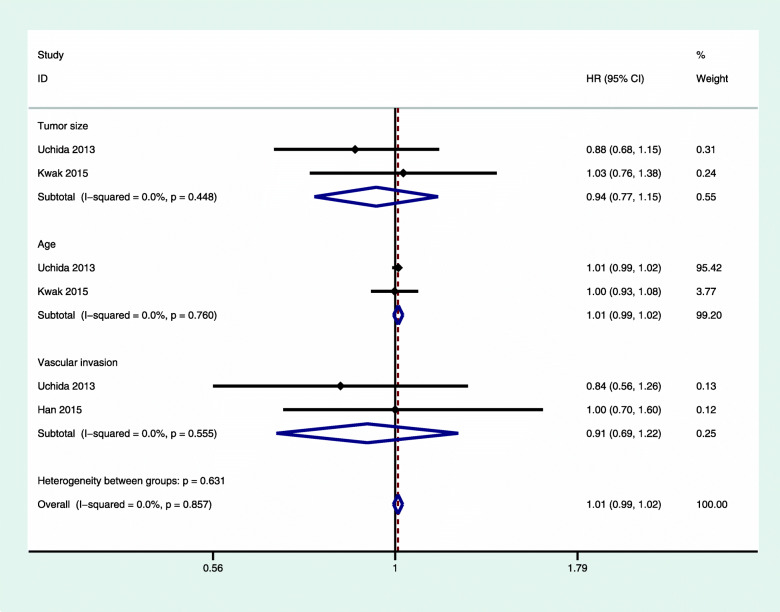
The subgroup analysis of the DFS/RFS of luminal A breast cancer with chemotherapy versus nonchemotherapy. CI, confidence interval; HR, hazard ratio; RFS, relapse-free survival; DFS, disease-free survival

We also performed a subgroup analysis for the following factors: age, tumor size, and vascular invasion. We did not find a significant correlation in any of these analyses, and we did not observe any significant heterogeneity (Figs. 6 and 7).

### Sensitivity analysis and publication bias

**Fig. 8:**
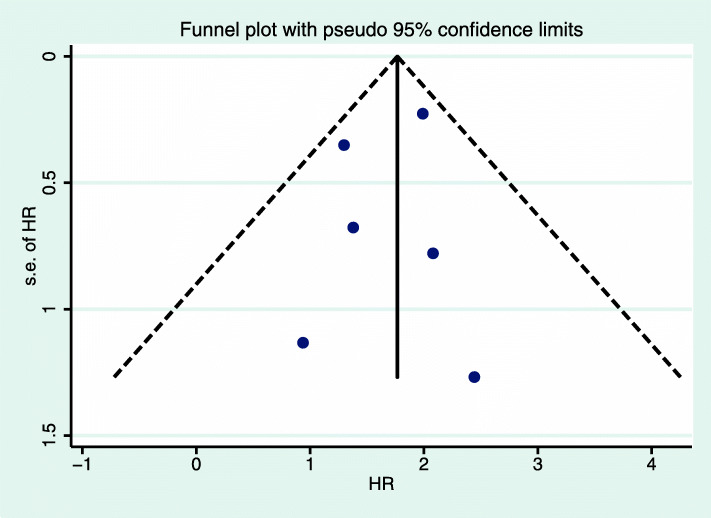
Funnel plot of OS of luminal A breast cancer with pseudo 95% confidence limits. HR, hazard ratio; OS, overall survival

**Fig. 9:**
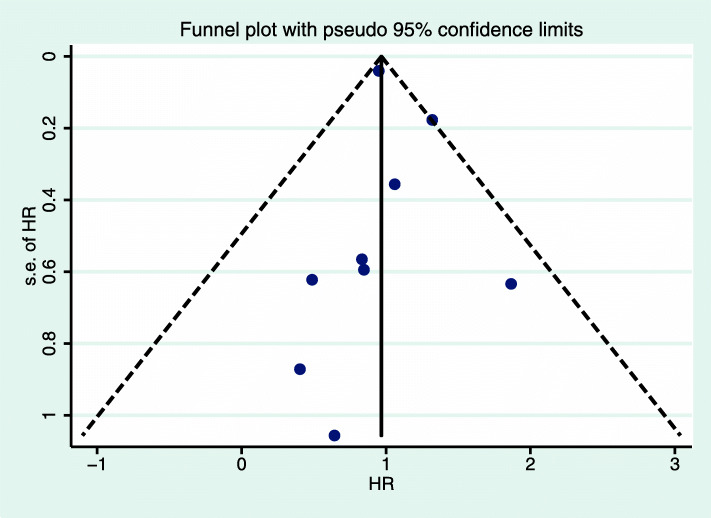
Funnel plot of DFS/RFS of luminal A breast cancer with pseudo 95% confidence limits. HR, hazard ratio; RFS, relapse-free survival; DFS, disease-free survival

We conducted a sensitivity analysis and found that no study had an excessive effect on the relationship between the prognosis of luminal A breast cancer and chemotherapy. The funnel chart (Figs. 8 and 9) shows that the prognosis of luminal A breast cancer patients was related to chemotherapy (OS: Egger, *P* = 0.70; Begg, *P* = 0.61; DFS/RFS: Egger, *P* = 0.70; Begg, *P* = 0.75). The analysis did not reveal any publication bias.

## Discussion

Chemotherapy is an important treatment for breast cancer. Adjuvant chemotherapy can reduce the recurrence rate [21]. However, because of tumor heterogeneity, chemotherapy of different breast cancer molecular subtypes has different effects [3]. We found that chemotherapy did not improve the OS of patients with luminal A subtype breast cancer (1.73, 95% CI 1.23, 2.43). Interestingly, patients who received only endocrine therapy had better prognosis compared with patients who received both chemotherapy and endocrine therapy, regardless of whether the lymph nodes metastasized (Figs. 2 and 4). The RFS/DFS of luminal A breast cancer patients did not show significant associations, which indicated that chemotherapy did not reduce the risk of disease recurrence.

We analyzed the reasons for these results from several aspects. First, not all lymph node-positive luminal A subtype breast cancer patients are suitable for chemotherapy. The American Society of Clinical Oncology guidelines indicate that low-risk, node-negative breast cancer patients will not benefit from chemotherapy [22]. In addition, Han et al. reported that when the number of positive lymph nodes was ≥ 4 patients should receive chemotherapy [17]. Herr et al. verified this conclusion. When the number of lymph node positives was ≥ 4, the OS of patients after chemotherapy improved, whereas 1–3 lymph node-positive patients with luminal A breast cancer did not have improved prognosis after chemotherapy [20]. The National Surgical Adjuvant Breast and Bowel Project B20 and Southwest Oncology Group 8814 found that not all patients with lymph node-positive luminal A subtype breast cancer benefited from chemotherapy. Both studies showed that Oncotype DX 21-gene recurrence score determined whether chemotherapy was needed [23, 24]. The Southwest Oncology Group 8814 study showed that postmenopausal women with lymph node-positive luminal A subtype breast cancer with low (< 18) or moderate (18 < RS < 31) recurrence scores did not benefit from chemotherapy [23]. Park et al. reported that chemotherapy did not significantly improve the prognosis of patients with luminal A, lymph node-positive breast cancer [25].

Second, the side effects of chemotherapy negatively affect prognosis. Older patients have diminished tolerance to chemotherapy. In a clinical trial, Muss et al. showed that, compared with patients aged ≤ 50 years (< 0.001), patients aged > 65 years who received chemotherapy had higher likelihoods of developing grade 4 hematologic toxicity [26]. Older patients have a higher prevalence of comorbidities and functional decline. These conditions impair survival, especially survival of patients who received chemotherapy [27, 28]. For elderly patients, the side effects of chemotherapy cancel the benefits.

Regarding the relationship between lymph node positivity and breast cancer prognosis, in some cases, lymph node positivity will affect the prognosis of breast cancer. For example, when the lymph node is positive, extra-lymph node invasion is an important factor that affects prognosis. Some investigators found that the poor prognosis may be related to the invasion of tumor cells into adipose tissue around lymph nodes [29–31]. In addition, tumor markers can also predict the prognosis of lymph node metastasis in breast cancer. The silent mating type information regulation 2 homolog 1, a tumor suppressor, can inhibit lymph node metastasis by activating cysteine aspartate protease 3 [32]. However, breast cancer prognosis is not always related to lymph node positivity. Shigematsu et al. found that when the sentinel lymph node metastasis was detected in patients with cT1-2N0M0 lymph node metastasis did not affect the RFS [33].

Age is a factor in the treatment of breast cancer because age is associated with the effectiveness and tolerance of chemotherapy drugs. Delgado-Ramos et al. showed that chemotherapy could be used effectively in older women with early-stage breast cancer, and chemotherapy was well-tolerated, with few life-threatening or fatal toxic reactions [27]. Haque et al. found that people aged > 50 were more likely to benefit from chemotherapy and have longer OS [34]. Contrary to the Delgado-Ramos study, we found that age did not affect the relationship between prognosis of luminal A, lymph node-positive patients and chemotherapy. This difference may be related to molecular typing, and we need more research to test that idea.

Tumor size is often associated with prognosis. Kustic et al. showed that, in nonluminal A breast cancer, large tumor size was associated with poor prognosis and adversely affected DFS and OS [35]. In luminal type A breast cancer, many studies have failed to find association between tumor size and prognosis, regardless of lymph node positivity [10, 19, 20]. Similarly, we did not find association between tumor size and prognosis.

Vascular invasion is another factor that influences the course of breast cancer. In a meta-analysis, Zhang et al. showed that lymphatic vessel invasion was a predictor of lymph node metastasis, and peritumoral vessels and lymphatic vessels were important means of lymph node metastasis [36]. We assessed vascular invasion and found that, in luminal A, lymph node-positive breast cancer, vascular invasion did not significantly affect the relationship between chemotherapy and breast cancer prognosis.

Our study had some limitations. Although we checked for heterogeneity and publication bias to ensure the accuracy of the study, more research is needed because of the small number of articles that we surveyed. In addition, we did not analyze the effect of the number of positive lymph nodes on prognosis. Further study is needed on this issue as well.

## Conclusion

We found that chemotherapy for luminal type A, lymph node-positive breast cancer did not improve OS or RFS/DFS. Instead, the side effects of additional chemotherapy may jeopardize prognosis. Whether the number of positive lymph nodes also affects the outcome of chemotherapy remains to be assessed with prospective studies.

## Data Availability

The datasets generated and/or analyzed during the current study are available in the published articles cited in the text.

## Abbreviations

ER: Estrogen receptor
PR: Progesterone receptor
HER2: Human epidermis growth factor receptor-2
NCCN: The National Comprehensive Cancer Network
OS: Overall survival
RFS: Relapse-free survival
DFS: Disease-free survival
RR: Relative risk
HR: Hazard ratio
OR: Odds ratio
CI: Confidence interval
PRISMA: Preferred Reporting Items for Systematic Reviews and Meta-Analyses
F: Fluorouracil
E: Epirubicin
C: Cyclophosphamide
A: Anthracycline
M: Methotrexate
T: Taxane
SIRT1: The silent mating type information regulation 2 homolog 1
